# Report from the 2026 Barth Syndrome Foundation International Scientific, Medical & Family Conference: Advances in Barth Syndrome Research, Therapy Development, and Clinical Care

**DOI:** 10.3390/jdb14040045

**Published:** 2026-10-19

**Authors:** Lindsay Marjoram

**Affiliations:** Barth Syndrome Foundation, 2005 Palmer Avenue #1033, Larchmont, NY 10538, USA; lindsay.marjoram@barthsyndrome.org; Tel.: +1-914-303-6323

**Keywords:** Barth syndrome, TAFAZZIN, cardiolipin, ABHD18, elamipretide, gene therapy, enzyme replacement therapy, mitochondrial disease, ultra-rare disease drug development, natural history, Centers of Expertise

## Abstract

The 2026 Barth Syndrome International Scientific, Medical & Family Conference was held at the Hyatt Regency Coconut Point, Bonita Springs, Florida, USA, from 19th to 25th July 2026 with scientific programming beginning 22nd July. This biennial event assembled researchers, clinicians, and affected community members for its first gathering since the U.S. FDA granted accelerated approval to elamipretide (Forzinity™; Mighty Therapeutics), the first therapy for Barth syndrome and first ever mitochondrially targeted therapy, in September 2025. Scientific sessions addressed: (1) emerging cardiolipin biology; (2) convergent identification of ABHD18 as a therapeutic target for correction of the cardiolipin phenotype; (3) advancing therapeutic strategies including AAV gene therapy, enzyme replacement therapy, and ABHD18 inhibition; (4) the elamipretide post-approval program, including the SPIBA-401 confirmatory trial; (5) natural history characterization covering infant outcomes, extra-cardiac manifestations, diaphragm weakness, neutropenia, and amino acid metabolism; (6) regulatory and drug development strategies in ultra-rare indications; and (7) international Centers of Expertise models and the launch of the BarthINSIGHTS registry. This report summarizes the key scientific advances, discussions, and future priorities as they relate to Barth syndrome biology, therapeutic development, and clinical care.

## 1. Introduction

Barth syndrome (BTHS; OMIM 302060) is an X-linked mitochondrial disorder caused by pathogenic variants in the gene *TAFAZZIN*, which encodes a mitochondrial transacylase essential for the remodeling of cardiolipin (CL)—the signature phospholipid of the inner mitochondrial membrane [1]. *TAFAZZIN* loss of function results in accumulation of monolysocardiolipin (MLCL) and depletion of mature tetralinoleoyl CL; mass spectrometric measurement of the MLCL:CL ratio is a sensitive and specific method for diagnosing BTHS or confirming the pathogenicity of a variant of unknown significance [2,3]. The mitochondrial bioenergetic deficits in BTHS lead to a multisystem disorder characterized by dilated cardiomyopathy, skeletal muscle myopathy, neutropenia, growth delays, gastrointestinal dysfunction, and exercise intolerance [4,5].

The 2026 Barth Syndrome International Scientific, Medical, and Family conference was hosted by the Barth Syndrome Foundation (BSF) and convened from 19th to 25th July 2026 in Bonita Springs, Florida. This unique event brings together individuals affected by BTHS and their extended families as well as clinical providers and scientists for a weeklong event of programming tailored to each group. The first two and a half days began with programming for affected individuals and their families and concurrent IRB-approved on-site clinical and human subjects research ranging from survey-based studies to functional measures of movement, echocardiograms, and biospecimen collections including blood and fecal material. This conference is the largest gathering of individuals affected by BTHS worldwide and provides an invaluable opportunity for researchers to enroll larger numbers of affected individuals in a given study while granting patients the opportunity to participate in several studies without having to travel to multiple locations. Over 300 participants from 11 different countries attended the event, representing a wide range of scientific backgrounds and reinforcing the idea that perturbations in CL synthesis have far-reaching impacts from developmental biology to translational drug discovery. Importantly, this conference was also the first gathering of the broader Barth community since the landmark FDA approval of elamipretide (Forzinity™; Mighty Therapeutics) in September 2025. Because elamipretide is the first therapy developed specifically for BTHS *and* the first mitochondrial-targeting therapy to be approved, this conference represented both a scientific milestone and an inflection point: confirmation of clinical benefit is required by regulatory mandate, a new generation of therapeutic approaches is entering preclinical development, and the clinical infrastructure needed to support future trials—validated outcome measures, natural history data, and international registry infrastructure—is rapidly evolving. The scientific program included invited platform talks, competitively selected research spotlight talks, two poster sessions, the rollout of BSF’s new registry/repository BarthINSIGHTS, a Centers of Expertise/Excellence (COE) working session, and plenary sessions devoted to invited perspectives on novel funding mechanisms, drug approvals, and regulatory considerations in ultra-rare drug development from leaders across the rare disease ecosystem.

The scientific and medical (SciMed) portion of programming opened with a popular segment called “Science Speed Dating” where five pre-selected scientific attendees each presented their lab’s work in lay-friendly terms to tables of affected family attendees, switching to a new table of families every 10 min. Following this session, the roles were reversed, and seven tables were led by affected individuals or caregivers, where each table focused on a topic central to BTHS life: infancy, aging, fatigue, feeding challenges, heart failure, neutropenia, and heart transplant. SciMed attendees visited each table for eight minutes before moving onto the next, engaging with and learning directly from affected individuals about their lived experience. Both sessions strengthened the bond between the affected and SciMed communities in an informal setting that facilitated open discussion and planted the seed for future interactions throughout the week and beyond. The SciMed sessions were structured around key themes, including the basic biology underlying BTHS, emerging therapies, clinical outcomes, novel research tools and strategies. In addition to the core programming, there were two poster sessions (one for SciMed, and one geared towards families), as well as three social and networking events, and the event concluded with the famous “Friday Night Social”, which further fostered community exchanges and gave space for new collaborations among attendees.

One hallmark of this event was providing professional development opportunities in which trainee-level scientists and clinical healthcare professionals could engage with established researchers and clinicians. Graduate students and postdoctoral fellows assisted in planning the conference, and a mentoring lunch was held at which all graduate/medical students and postdoctoral/medical fellows in attendance were paired up with established investigators from academia and industry to learn about future career paths. Trainees were also invited to apply for competitive slots for talks and posters, with six trainees selected for talks and 13 trainees selected for poster presentations. The conference successfully delivered rigorous scientific programming within a collaborative and inclusive patient-centered environment, making this conference the premier event for Barth syndrome research globally.

## 2. Emerging Voices: Presentations from the Next Generation of Barth Researchers

*Structural Insights:* Graduate student Jose Guadalupe Rosas Jimenez (Max Planck Institute of Biophysics, Frankfurt, Germany) shared data evaluating a new cryo-electron microscopy structure of TAFAZZIN, utilizing AlphaFold3 and molecular dynamics modeling. His structural findings provide critical insights into how several known pathogenic mutations impact TAFAZZIN protein function [6]. *Molecular Underpinnings:* Abu Ramim, a new postdoctoral fellow at UT Southwestern (Dallas, TX, USA), presented data from the culmination of his graduate work in Dr. Miriam Greenberg’s lab at Wayne State University (Detroit, MI, USA). Using *TAFAZZIN*-deficient mouse myoblasts, Ramim observed increased proline catabolism for the TCA cycle and a concomitant loss of proline from collagen, which impaired collagen synthesis and extracellular matrix remodeling [7]. *Muscle Impact:* Sree Venigalla, a postdoctoral researcher in Dr. Christina Pacak’s lab (University of Minnesota, Minneapolis, MN, USA), presented work on the effects of extracellular matrix in BTHS using 3D engineered heart tissues by co-culturing BTHS or healthy induced pluripotent stem cell (iPSC)-derived cardiac fibroblasts and cardiomyocytes. The team found that BTHS-derived fibroblasts impacted contractile force and mitochondrial function even in healthy cardiomyocytes and that BTHS-fibroblast engineered heart tissues displayed a reduction in stiffness and reduced respiratory chain activity, suggesting that resident fibroblasts are active drivers of pathology. *Genomic Landscape:* Recent college graduate and research assistant Chaeyoung (Jennifer) Park (Boston Children’s Hospital, Boston, MA, USA) presented her work from Dr. William Pu’s lab, which aims to further our understanding of how modifier genes impact the severity of BTHS [8]. Diversity outbred mice, developed at The Jackson Laboratory (Bar Harbor, ME, USA), were bred with *TAFAZZIN* heterozygous females on the C57BL/6J background to produce 200 *TAFAZZIN* null males on different genetic backgrounds. There was a broad spectrum of cardiac and exercise phenotypes ranging from normal to significantly impacted. Linkage analysis identified significant linkage to key phenotypes including neutrophil count, heart weight, and treadmill endurance. Efforts are now underway to pinpoint these modifier genes and understand their mechanisms of action.

## 3. Cardiolipin and TAFAZZIN Biology

Three of the conference speakers presented data that broadened the field’s understanding of how CL deficiency leads to disease pathology at a molecular level. Itay Budin (UCSD, San Diego, CA, USA; 2026 BSF grant awardee) presented his lab’s findings that the pathological severity of *TAFAZZIN* deficiency is modulated by the fatty acid saturation of the surrounding phospholipid environment. In yeast *TAFAZZIN* knockout (KO) systems, microaerobic conditions—which favor lipid saturation—dramatically amplified the MLCL:CL phenotype, establishing molecular oxygen as an electron acceptor for the desaturation enzymes that maintain CL unsaturation [9]. Fabian Finger (University of Copenhagen, Copenhagen, Denmark) reported that cardiolipin synthase (CRLS1) expression and CL abundance decline specifically in glycolytic skeletal muscle fibers with age, with conditional Crls1 knockdown in mice producing selective glycolytic fiber atrophy and a whole-body shift toward increased glucose utilization—a metabolic remodeling consistent with the fatigue and exercise intolerance characteristic of adult BTHS patients [10]. Seul Kee Byeon (Mayo Clinic, Rochester, MN, USA) presented a systematic lipidomic characterization of dried blood spot samples from female BTHS carriers, showing the first evidence that the MLCL:CL ratio of carrier females is readily distinguishable from non-carriers and indicating that there may be broader systemic differences between carriers and non-carriers.

Simon Conway (Indiana University, Indianapolis, IN, USA) recently developed a clinically relevant CRISPR knock-in mouse model, *TAZ^D75H^*, based on a pathogenic point mutation observed in a BTHS patient that disrupts TAFAZZIN’s enzymatic activity while preserving TAFAZZIN protein expression [11]. Systematic characterization of the *TAZ^D75H^* mouse by the Conway lab has revealed disease-associated abnormalities across multiple organ systems including the heart [11], skeletal muscle [12], diaphragm [13], testes [14], and hematopoietic/immune system [15]. In his conference presentation, Dr. Conway highlighted initial findings from bulk RNA-sequencing analyses on cardiac tissues from *TAZ^D75H^* mice at three stages: postnatal day 5, 4 weeks and 6 months. These stages were chosen strategically to identify molecular changes associated with the transition from early disease to a progressive cardiac pathology. At all stages, the team observed the mitochondrial Integrated Stress Response. Further, several candidate targets were broadly clustered into two categories: signaling molecules or cell adhesion molecules that were upregulated only in the adult hearts. The data suggest that altered intercellular signaling and/or cell–cell or cell–matrix interactions may contribute to disease progression, and ongoing studies are investigating these candidates individually and how they are impacted in the *TAZ^D75H^* model.

Seth Corey (Cleveland Clinic, Cleveland, OH, USA) presented the first CRISPR model of BTHS in zebrafish, which shared some cardinal characteristics with BTHS manifestations experienced by humans, including neutropenia and elevated MLCL:CL [16]. Although zebrafish have only one *TAFAZZIN* gene and maternal zygotic mutants displayed significant oxygen consumption deficits, the characteristic heart, growth, and mitochondrial ultrastructural phenotypes were not apparent in the zebrafish model. The team, which includes co-investigator Yana Sandlers (Cleveland State University, Cleveland, OH, USA), hypothesizes that there may be salvage mechanisms utilized in zebrafish that bypass the deleterious effects caused by *TAFAZZIN* loss of function; identification of this mechanism could provide new inroads for therapeutic intervention(s) in BTHS.

## 4. ABHD18: A New Therapeutic Target

Two recent publications from three independent research groups have identified α/β-hydrolase domain-containing protein 18 (ABHD18) as the mammalian CLD1 homolog responsible for deacylating nascent CL to MLCL. Inhibition of ABHD18 in a *TAFAZZIN* knockout mouse ameliorated the cardinal symptoms associated with Barth syndrome, establishing ABHD18 inhibition as a compelling therapeutic approach agnostic to patient genotype [17,18].

Mindong Ren (NYU Grossman School of Medicine, New York, NY, USA) presented work from his collaborative efforts with Michael Schlame (NYU Grossman School of Medicine) and Miriam Greenberg (Wayne State University) demonstrating that *ABHD18* knockdown in cells or inactivation in *Drosophila* resulted in an improved MLCL:CL ratio and increased CL levels [17]. Vincent Blomen (Scenic Biotech, Amsterdam, Netherlands) and Jason Moffat (SickKids, The Hospital for Sick Children, Toronto, Ontario, Canada) each presented follow-on work individually from their joint efforts showing identification of *ABHD18* through convergent genetic suppressor screens, which ultimately demonstrated that inhibition of *ABHD18* in *TAFAZZIN* null mice restored heart function and the MLCL:CL ratio to wildtype levels [18]. In partnership with Secarna (Planegg, Germany), Dr. Blomen showed proof-of-concept data for antisense oligonucleotide candidates against *ABHD18*, with in vivo efficacy studies ongoing. Dr. Moffat showed proof-of-concept data for small molecule-mediated inhibition of *ABHD18* in a *TAFAZZIN* knockdown zebrafish model and presented current efforts on small molecule discovery. Finally, Timothy Cardozo (NYU Grossman School of Medicine; 2026 BSF Grant Recipient) presented his lab’s efforts to utilize a computationally accelerated model for identification of ABHD18 small molecule inhibitors. Together, these studies highlight the significance of ABHD18 as a novel and druggable target for the treatment of BTHS that is agnostic to the disease-causing variant.

## 5. Emerging Therapies for Barth Syndrome

Updates on two advanced preclinical programs for TAFAZZIN supplementation were presented: AAV gene addition and enzyme replacement therapy. William Pu (Boston Children’s Hospital) reported updated data from a TAFAZZIN gene replacement program [19] using a myotropic AAV capsid driving TAFAZZIN. Using a conditional *TAFAZZIN* knockout mouse model [20], Dr. Pu demonstrated that treatment with AAV-TAFAZZIN restored cardiac ejection fraction, normalized the MLCL:CL ratio in heart and skeletal muscle, rescued mitochondrial cristae morphology, and improved neuromuscular performance over 12 weeks [21]. Michael Chin (Tufts University, Medford, MA, USA) demonstrated that weekly dosing of a TAFAZZIN enzyme replacement therapy (ERT) using recombinant TAFAZZIN fused to a cell-penetrating peptide [22] in *TAFAZZIN* null mice significantly improved the MLCL:CL ratio across a broad array of tissues, restored mitochondrial oxygen consumption and cristae morphology, and improved cardiac and skeletal muscle performance, without any detectable anti-TAFAZZIN antibodies [23].

In addition to TAFAZZIN-targeted approaches, two research groups presented emerging clinical-stage therapies that work independently of or downstream of TAFAZZIN to improve mitochondrial health. Takaaki Abe (Tohoku University Graduate School of Medicine, Sendai, Japan) reviewed data from his lab’s work on mitochonic acid 5 (MA-5), an oral drug that was shown to improve mitochondrial function in a BTHS patient-derived iPSC line and in a *Drosophila* model of BTHS [24]. The investigators have already completed a Phase 1 trial in Japan in healthy individuals and are now enrolling for a Phase 2 trial on mitochondrial disease patients impacted by hearing loss but are not yet evaluating this drug clinically for the treatment of Barth syndrome. Priscilla Thakoerdien (Amsterdam University Medical Center [AMC], Amsterdam, Netherlands) presented preclinical data on the vitamin B3 analog, nicotinamide riboside [25]. Like MA-5, this supplement works independently of TAFAZZIN and improves mitochondrial function and exercise capacity in a *Drosophila* model of BTHS. The research team, led by Dr. Riekelt Houtkooper, is now preparing for a clinical trial (NAD4BARTH) to evaluate the drug’s efficacy in a clinical trial with Barth-affected individuals.

## 6. Elamipretide: Post-Approval Program and Evidence Expansion

Anthony Abbruscato and Amy Meadowcroft (Mighty Therapeutics, Needham, MA, USA) presented a comprehensive update on the elamipretide (Forzinity™) clinical program. Elamipretide received FDA accelerated approval in September 2025 based on improvement in the intermediate clinical endpoint of knee extensor muscle strength. The required confirmatory Phase 3b/4 trial, SPIBA-401 (4TAZPOWER [26]), is an international 72-week randomized, double-blind, placebo-controlled study that will enroll ~48 patients aged ≥5 years. The primary endpoint will be a composite of three functional tests: 6 min walk test, triple timed up-and-go, and five times sit-to-stand. As of this writing, two patients have been enrolled at a clinical site in Bristol, UK, and an additional site in Canada has begun recruitment; additional international sites are anticipated in the coming months [27]. Concurrently, Mighty Therapeutics has aligned with FDA on a pediatric extrapolation framework for Barth-affected individuals weighing 7–30 kg to participate in an open-label Phase 1 pharmacokinetic study enrolling approximately six patients to support a supplemental new drug application to expand elamipretide’s label.

Two compelling case reports were presented that further extended the evidence base for elamipretide. Pascal Amedro (Children’s National Hospital, Washington, DC, USA) described the case of a 5-year-old child with Barth syndrome listed for heart transplant in France who received access to elamipretide via the Expanded Access Program (EAP) in Europe. Over six months of treatment, the child’s left ventricular ejection fraction improved from 20 to 50%; the child was delisted for transplant and was able to resume schooling [28]. Majid Husain (UCLA, Los Angeles, CA, USA) presented a case report on an infant diagnosed with BTHS who received EAP elamipretide beginning at three weeks of age, underwent heart transplant at five months of age, and continued to receive elamipretide post-transplant [29]. Twenty-four months post-transplant, the affected child was meeting developmental milestones consistent with other BTHS infants and had an unremarkable post-transplant course. Taken together in the context of previously published case reports on the use of elamipretide in children, these findings suggest a favorable side effect profile and consistent improvements in muscle strength [30,31,32]. The results from the Phase 4 confirmatory trial and future case reports will help regulators, scientists, and patients better understand the potential range and magnitude of clinical impacts of elamipretide on Barth-affected individuals.

## 7. Understanding Barth Syndrome Across the Lifespan

Priscilla Thakoerdien (AMC) presented on one of the newest BTHS COEs at AMC, which was supported by BSF and received formal accreditation from the Dutch government in December 2025, 42 years after Dr. Peter Barth first characterized the disease there [4]. The center has assembled a multidisciplinary team to provide comprehensive Barth care at AMC and has already identified additional affected individuals in the Netherlands and surrounding countries who were not previously known to BSF or AMC.

Hilary Vernon (Johns Hopkins Medicine/Kennedy Krieger Institute, Baltimore, MD, USA), who was awarded BSF’s top scientific honor of the Varner Pioneer in Science and Medicine Award [33] at the 2026 conference, presented longitudinal data from 21 BTHS patients enrolled in an observational natural history study from infancy [34]. The mean age at diagnosis was 5.5 months, reflecting the impact of expanded genetic testing panels. Cardiomyopathy was the presenting feature in half of the cases, with 18/21 ultimately being diagnosed with cardiomyopathy and a left ventricular ejection fraction averaging 26%; ~24% received heart transplants by the time of publication. Feeding challenges were pervasive (76%), further highlighting the pressing need to address this manifestation within the BTHS patient community [5]. Gross motor delays were universal for later milestones like standing and walking, and low quality-of-life scores on the PedsQL™ instrument suggest that the non-cardiac burden is grossly underestimated by current assessment approaches.

Understanding how BTHS impacts affected individuals across their lifespan and across the globe is a key agenda item for BSF, with particular focus on how the disorder impacts individuals as they age. Importantly, extra-cardiac manifestations in adults received significant attention during the conference. Pawel Pomianowski (University of Mississippi Medical Center, Jackson, MS, USA) reported on a newly formed COE at the University of Mississippi Medical Center and presented data from the first clinic visits which revealed an unanticipated prevalence of abnormal blood glucose and osteopenia among adult patients. These findings were not captured in standard cardiology notes and prompted the Mississippi COE to integrate an endocrinologist into their multidisciplinary clinic team. Vinicius Mariani (Duke University, Durham, NC, USA) presented preliminary evidence of diaphragm weakness as an under-recognized problem in BTHS patients; re-evaluation of exercise testing data from Dr. Todd Cade (Duke University; 2026 BSF Grant Recipient) identified ventilatory abnormalities in both pediatric and adult cohorts. Alongside his postdoctoral mentor Dr. Leonardo Ferreira (Duke University), Dr. Mariani conducted on-site clinical research at the conference to further evaluate inspiratory neuromuscular dysfunction in BTHS-affected individuals ages 3 and up, and in parallel, the Ferreira lab is conducting mouse experiments to investigate breathing and gas exchange in vivo and diaphragm muscle morphology and mechanics ex vivo.

In addition to understanding how BTHS impacts different body systems over time, a team of researchers, including BSF research and development staff, have begun evaluating the healthcare costs associated with BTHS [35]. Neurologist Mary Kay Koenig (McGovern Medical School, Houston, TX, USA) presented data from a recent publication which highlights the challenges of estimating healthcare costs in ultra-rare diseases. Using a combination of real-world evidence including claims datasets, the medical literature, and the BarthINSIGHTS patient registry data, we found that BTHS is, unsurprisingly, a costly condition associated with high healthcare resource utilization. Continued efforts to better define healthcare resource utilization are underway to provide higher-resolution insights into this complex disorder.

## 8. Learning from Others: Successful Approaches to Ultra-Rare Drug Development

During his conference keynote address, Emil Kakkis (Ultragenyx, Novato, CA, USA) delivered an analysis of the structural failures of current drug development frameworks for ultra-rare diseases, identifying four reforms that could improve the speed and cost of drug development: (1) redefining a disease by its root mechanistic cause rather than by specific clinical outcomes; (2) replacing single, primary endpoint, randomized control trial designs with multi-domain composite endpoints; (3) changing chemistry, manufacturing and controls (i.e., CMC) requirements to reflect the scale of the patient population; and (4) restoring age-agnostic indication labeling for ultra-rare diseases—a standard reversed in recent years.

The conference plenary session included three segments: (1) Novel Paths to Drug Approval in Rare Disease, (2) Novel Therapeutic Modalities for Rare Disease, and (3) New Regulatory Considerations for Rare Disease. In the first session, Eric Hoffman (ReveraGen; Binghamton University, Binghamton, NY, USA), Cassi Friday (CureHHT, Monkton, MD, USA), and Terry Pirovolakis (Elpida Therapeutics, Encino, CA, USA) presented lessons learned that were directly applicable to BTHS: baseline infrastructure including natural history and endpoints must be in place to support future clinical trials; natural history data that are not regulatory-grade cannot support trial design; and registry-first approaches can be used to build the critical knowledge base needed for clinical success. In the second session, Rebecca Ahrens-Nicklas (Children’s Hospital of Philadelphia, Philadelphia, PA, USA), Patrick Gonzalez (Solid Biosciences, Charlestown, MA, USA), and Scott Henry (Ionis Pharmaceuticals, Carlsbad, CA, USA) discussed recent clinical experiences with base editing [36], AAV-mediated gene therapy [37], and antisense oligonucleotides [38], respectively. Each speaker demonstrated meaningful clinical progress while also being candid about the constraints of each platform. While single-patient (i.e., N-of-1) case reports are compelling, Dr. Ahrens-Nicklas highlighted that they are not scalable to treat larger populations. All three speakers identified patient-centered outcome measures as an underutilized opportunity. The emerging FDA plausible mechanism framework was described [39,40] by the panelists as genuinely promising but, in its current iteration, inaccessible for most small organizations, as it requires entering directly into a biologics licensing application (BLA)-enabling trial with CMC fully locked—a financial bar far in excess of what most patient advocacy organizations can support. Across all three platforms, speakers encouraged drug developers to engage FDA early and concretely to build a shared regulatory understanding before a pivotal trial begins. In the final segment of the plenary session, Janet Woodcock (formerly FDA Center for Drug Evaluation and Research) and Amy Rick (FDA Rare Disease Innovation Hub or RDIH) discussed ways in which ultra-rare indications can succeed within existing regulatory frameworks. Like Emil Kakkis, Janet Woodcock emphasized that the level of requirements and expectations should be proportionate to the patient population rather than a one-size-fits-all approach. She also stressed that researchers must know as much as possible about the disease before the start of the pivotal trial, including natural history, dosing and tolerability, effect size of the treatment and timescale of the treatment, which means preliminary clinical work is necessary and required. Amy Rick explained the goals of the newly formed RDIH at FDA which are both internally (e.g., cross-center information sharing and alignment) and externally facing (e.g., stakeholder engagement including patient advocacy organizations). Both Woodcock and Rick identified the development of rigorous, patient-defined outcome measures as the highest-value scientific investment that patient advocacy organizations like BSF can make.

## 9. Special Working Sessions

The Barth Syndrome International Conference represents a unique opportunity to hold in-person working sessions with diverse stakeholders that share a vested interest in improving the lives of those living with BTHS. To this end, two working sessions warrant a brief spotlight. In the first session, representatives from all established COEs including Kennedy Krieger Institute (Baltimore, MD, USA), University of Mississippi Medical Center (Jackson, MS, USA), National Health Service (Bristol, United Kingdom), and AMC (Amsterdam, Netherlands) as well as medical centers with a deep foundation in BTHS clinical care (e.g., France and Italy) met for a two-hour session devoted to the following agenda items: (1) establishing minimum components of a BTHS COE, (2) unanticipated hurdles when building a new COE, (3) core criteria to implement a new COE, and (4) recent learnings from each center. The intended outcome is to distill these shared learnings into a guidance document that helps inform where opening new COEs may be appropriate and what the minimum viable product is for a COE such that (1) comprehensive expert-level care is available to BTHS-affected individuals regardless of geography and (2) standardized data collection continues to expand the BTHS natural history.

In the second working session, which was held in hybrid format with live translation, leadership from BSF and its international affiliates met to discuss the launch of BarthINSIGHTS [41], BSF’s newly updated and rebranded patient registry and repository. Building on the registry data that BSF has collected since 2014, BarthINSIGHTS is a modern, nimble, and secure database that collects patient-centered data on the diagnostic odyssey, manifestations, and disease progressions of BTHS to inform regulatory pathways for drug development, and demonstrates the unmet needs of our global community, while simultaneously sharing insights directly back to participating families. A global unique identifier (GUID) is associated with each validated patient, enabling integration with external research programs and associated biospecimens, which BSF maintains in a biorepository. BarthINSIGHTS is updated iteratively and responsively to community needs, with language translations and electronic medical record integration representing the next enhancements to the system.

## 10. Future Directions and Research Priorities

The scientific program highlighted several high-priority research directions that will continue to redefine the field over the coming years. ABHD18 inhibition—via antisense oligonucleotides, small molecules, or other modalities—represents an immediately actionable, next-generation therapeutic target, with in vivo readouts from several programs anticipated in 2027. Clinically, the global SPIBA-401 confirmatory trial by Mighty Therapeutics and the NAD4BARTH trial at AMC are providing a level of opportunity not yet experienced by the BTHS community. Across all therapeutic programs, the development and validation of Barth-specific, patient-defined functional outcome measures and biomarkers emerged as a pressing need. Simultaneously, the field must continue to bolster the regulatory-grade natural history infrastructure that emerging programs will require. Expansion of the international COE network, guided by a framework to be developed by the COE working group, and continued growth of BarthINSIGHTS will be essential to ensure that expert BTHS care can be accessed around the globe.

## 11. Conclusions

The 2026 Barth Syndrome International Scientific, Medical & Family Conference documented a field at a genuine inflection point: for the first time, BTHS has an approved therapy, multiple next-generation programs in advanced preclinical development, a growing international clinical infrastructure, and a patient registry/repository designed from the ground up to support regulatory-grade evidence generation by researchers worldwide. The convergence of independent research groups on ABHD18 as a druggable, *TAFAZZIN*-agnostic target is one of the most significant findings for BTHS in the past several years, and the breadth of therapeutic modalities now in active development—gene therapy, enzyme replacement, ABHD18 inhibition, and NAD precursor supplementation—reflects the maturation of a field that was, until very recently, without any approved treatment. Yet, there is still much work to do: the clinical infrastructure must keep pace with the science, and the endpoints, natural history data, and international architecture needed to support the next generation of trials must be built now. BSF is uniquely poised to serve as a partner in this work, and the community-centered model of this conference—in which affected individuals, caregivers, clinicians, and scientists share the same space, inform each other’s priorities, and build durable collaborations—remains the field’s most distinctive and irreplaceable asset.

## Data Availability

No new data were created or analyzed in this study. Data sharing is not applicable to this article.

## Abbreviations

The following abbreviations are used in this manuscript:

BTHS	Barth syndrome
CL	Cardiolipin
MLCL	Monolysocardiolipin
BSF	Barth Syndrome Foundation
COE	Center of Expertise/Excellence
SciMed	Scientific and Medical
iPSC	Induced pluripotent stem cell
KO	Knockout
CRLS1	Cardiolipin Synthase
ABHD18	α/β-hydrolase Domain-containing Protein 18
ERT	Enzyme Replacement Therapy
MA-5	Mitochonic Acid 5
AMC	Amsterdam University Medical Center
EAP	Expanded Access Program
CMC	Chemistry, Manufacturing, and Controls
BLA	Biologics Licensing Application
RDIH	Rare Disease Innovation Hub
GUID	Global Unique IDentifier
